# Psychometric properties of a custom Patient-Reported Outcomes Measurement Information System (PROMIS) physical function short form and worst stiffness numeric rating scale in tenosynovial giant cell tumors

**DOI:** 10.1186/s41687-020-00217-6

**Published:** 2020-07-16

**Authors:** Rebecca M. Speck, Xin Ye, Nicholas M. Bernthal, Heather L. Gelhorn

**Affiliations:** 1 Evidera, 7101 Wisconsin Ave, Suite 1400, Bethesda, MD 20814 USA; 2 Daiichi Sankyo, Inc., 211 Mount Airy Rd, Basking Ridge, NJ 07920 USA; 3 Department of Orthopaedic Surgery, University of California, Los Angeles, 615 Charles E. Young Dr. South Rm 410, Los Angeles, CA 90095 USA

**Keywords:** TGCT, Psychometrics, Patient-reported outcomes, PROMIS, Worst stiffness NRS

## Abstract

**Purpose:**

The purpose of this study was to evaluate the psychometric properties of the PROMIS-Physical Function (PF) and Worst Stiffness Numeric Rating Scale (NRS) among patients with tenosynovial giant cell tumors (TGCT).

**Methods:**

Measurement properties of the customized lower extremity (LE) and upper extremity (UE) PROMIS-PF scales and Worst Stiffness NRS were assessed using data from the Phase 3 ENLIVEN trial (*n* = 120). Anchor- and distribution-based analyses were utilized to derive a responder threshold for meaningful change over time. The Patient Global Rating of Concept (PGRC)-Physical Functioning and Patient Global Impression of Change (PGIC)-Stiffness served as anchors. Responsiveness and responder threshold analyses were from baseline to week 25.

**Results:**

Cronbach’s alpha values for internal consistency reliability were 0.93 and 0.91 for the PROMIS-PF LE and UE, respectively. Test-retest reliability intra-class correlation coefficients were > 0.75 for both instruments. Convergent validity for both instruments was supported by moderate to strong correlations (≥0.30) with the Brief Pain Inventory and EQ-5D. Known-groups validity was established between subgroups stratified by pain level (*p* < 0.05). Responsiveness was supported by evaluating change scores among different levels of change in PGRC-Physical Functioning and PGIC-Stiffness (overall F values < 0.001). Triangulation of responder definition analyses resulted in a threshold of ≥3 for the PROMIS-PF and ≥ 1 for the Worst Stiffness NRS.

**Conclusion:**

This study is the first to establish the psychometric properties of patient-reported outcome measures in TGCT. The evidence demonstrates that the PROMIS-PF and Worst Stiffness NRS have good reliability, validity, and responsiveness, and provides guidance for the interpretation of meaningful change.

## Background

Tenosynovial giant cell tumors (TGCT) are rare non-malignant neoplasms that involve the synovium or tendon sheath. They typically present in young and middle-aged adults of both sexes, and result in functional limitations, morbidity, and decreased quality of life (QOL) [21]. Symptoms often include pain, stiffness, swelling, and reduced range of motion (ROM) of the affected joint. TGCT can be subdivided into 2 main subtypes: localized and diffuse, with localized presenting as a single nodule and diffuse presenting as an infiltrative, locally aggressive tumor. The main treatment option for TGCT is surgery, but diffuse disease can be challenging to manage surgically and recurrence rates are high (8%–56%) [14], so systemic anti-tumor agent options are of interest.

In the recently completed ENLIVEN Phase 3 trial (NCT02371369), pexidartinib, a novel, orally active, small molecule receptor tyrosine kinase inhibitor has demonstrated efficacy in reducing tumor size and improving functional outcomes [18]. The overall response rate per Response Evaluation Criteria in Solid Tumors version 1.1 (RECIST v1.1) [5] and TVS was 39% vs. 0% and 56% vs. 0% in pexidartinib and placebo patients, respectively [18]. Patient-reported physical functioning and stiffness were included in the trial as key secondary endpoints. Unlike many oncologic diseases, TGCT is non-fatal; thus, functional disability due to disease and improvement of physical function on therapy were considered of seminal importance. Through qualitative work completed in preparation of the ENLIVEN trial, which included interviews with both patients and clinicians, it was demonstrated that physical functioning and stiffness were important treatment outcomes to patients with TGCT [9].

Despite the importance of physical functioning and stiffness as outcomes in the treatment of TGCT, there were no PRO measures specific to this population available for inclusion in the ENLIVEN trial. Therefore, based on the qualitative work [9, 10], items from the Patient-Reported Outcomes Measurement Information System Physical Function (PROMIS-PF) item bank [1, 17] were included in the ENLIVEN trial to assess physical functioning. In addition, a single-item Worst Stiffness NRS was developed to assess stiffness. While the content validity of these items has been established in the TGCT patient population [9, 10], the psychometric properties, including item performance, reliability, validity, ability to detect change, and identification of a responder definition threshold, have yet to be demonstrated. This psychometric evidence is integral to having robust, valid, and reliable PRO measures for use in future clinical trials of therapies for TGCT. Therefore, the purpose of the current work was to describe the methods and present the results of the psychometric evaluation of the PROMIS-PF and Worst Stiffness NRS using data from the ENLIVEN trial.

## Methods

### Patients

ENLIVEN was a 2-part, multi-center, double-blind, randomized, placebo-controlled Phase 3 study designed to compare the response rate of pexidartinib with that of placebo per RECIST 1.1 at Week 25 in subjects with symptomatic TGCT for whom surgical resection would be associated with potentially worsening functional limitation or severe morbidity (locally advanced disease) [18]. In Part 1, the double-blind phase, eligible candidates were enrolled from May 11, 2015, to September 30, 2016, and centrally randomized in a 1:1 ratio to receive either pexidartinib or placebo for 24 weeks. Randomization was stratified by United States (US) versus non-US sites and by upper extremity (UE) versus lower extremity (LE) involvement.

Eligible patients were age 18 or older, had a histologically confirmed TGCT diagnosis, and had advanced disease for which surgical resection would be associated with potentially worsening functional limitation or severe morbidity. They had symptomatic disease defined as a worst pain or worst stiffness score of at least 4 at any time during the week preceding the Screening Visit (based on scale of 0 to 10, with 10 representing “pain as bad as you can imagine” or “stiffness as bad as you can imagine”), and measurable disease per RECIST v1.1 with a minimum size of 2 cm. 120 subjects across approximately 45 study sites in the US, Canada, EU, and Australia were treated, 61 with pexidartinib and 59 with placebo.

### Instruments

#### PROMIS-PF

Items from the validated PROMIS-PF item bank, which was designed to assess mobility, dexterity, axial, and complex activity function irrespective of specific anatomic location or acuteness of disease [1, 17], were used to assess physical functioning. Due to the heterogeneity in the physical impacts based on the tumor location, items for two customized tumor location-specific scales were selected based on input directly from patients on which activities were impacted by their TGCT [9, 10]. From the 121 validated items available, a 13-item scale and 11-item scale were customized to assess physical function among patients with tumors in the LE and UE, respectively. Nine of the PROMIS-PF items were overlapping across the two customized forms (i.e., included in both LE and UE scales).

Each PROMIS-PF question had five response options ranging in value from 1 to 5. Item-response theory-based parameters were used to calculate person-specific scores. A fixed-parameter calibration with no estimation was done using subject’s responses to the PROMIS-PF items to estimate person latent trait scores. Missing items were not imputed. The item parameters used to estimate person-latent trait scores were obtained from the PROMIS Assessment Center (https://www.assessmentcenter.net/). As is customary for PROMIS, the results are reported as T-scores, which represents physical functioning as a standardized score with a mean of 50 and a standard deviation (SD) of 10. A higher PROMIS T-score represents more of the concept being measured. For positively-worded concepts like physical function, a T-score of 60 is one SD better than average, and a person with a T-score of 40 is one SD worse than the average.

#### Worst stiffness NRS

The Worst Stiffness NRS was a single-item, which stated, “The following question asks about stiffness at the site of your tumor. Please rate your stiffness by circling the one number that best describes your stiffness at its worst in the last 24 hours.” For consistency the item had a response scale similar to that of the Brief Pain Inventory (BPI) Worst Pain NRS item [2, 4], that was a 0–10 NRS where zero is “no stiffness” and 10 was “stiffness as bad as you can imagine.” The item was included in ENLIVEN because qualitative interviews with patients and clinicians demonstrated that stiffness was an important treatment outcome [9].

The stiffness score was calculated using the number on the 11-point NRS selected by the patient for each day. The range for the score was 0 to 10. The weekly score was calculated as the average of non-missing records during each seven-day period, where the patient-reported entries on an outpatient basis were completed in at least 4 of the 7 days. (i.e., Mean weekly score = [sum of daily scores/# diary days completed]). Patients with fewer than 4 days of Worst Stiffness NRS entries had their stiffness scores for the week set to missing.

#### Other measures

The BPI Worst Pain NRS administered in ENLIVEN was a single-item, which stated, “The following question asks about pain at the site of your tumor. Please rate your pain by selecting the one number that best describes your pain at its worst in the last 24 hours.” The item was adapted from item 3 of the BPI-short form [2, 4] to include “pain at the site of your tumor.” The item has a response scale that is a 0–10 NRS where zero is “no pain” and 10 is “pain as bad as you can imagine.”

The EQ-5D-5L (heretofore referred to as EQ-5D) is a standardized measure of health status developed by the EuroQol Group in order to provide a simple, generic measure of health for clinical and economic appraisal [6]. The EQ-5D descriptive system includes five dimensions: mobility, self-care, usual activities, pain/discomfort and anxiety/depression. Each dimension has five levels: no problems, slight problems, moderate problems, severe problems and unable to/extreme. The EQ visual analogue scale (VAS) records the respondent’s self-rated health on a vertical VAS from 0 to 100 where the endpoints are labeled “Best imaginable health state (100)” and “Worst imaginable health state (0).”

The Patient Global Rating of Concept (PGRC)- Physical Functioning item was a single item that assessed the subject’s perception of physical functioning. Subjects were asked to indicate how much their tumor limits their ability to carry out every day physical activities on a 5-point Likert scale from “Not at all” to “Extremely”.

The Patient Global Impression of Change (PGIC) – Stiffness was a single item that assessed the subject’s perception of change in stiffness at the site of their tumor. Subjects were asked to indicate how much the stiffness at the site of their tumor had changed at Week 25 from Baseline on a 7-point Likert scale from “Much improved” to “Much worse.”

The Tumor Volume Score (TVS) was a semi-quantitative magnetic resonance imaging (MRI) scoring system that described tumor mass. The TVS was based on 10% increments of the estimated volume of the maximally distended synovial cavity or tendon sheath involved. Thus, a tumor that was equal to the volume of a maximally distended synovial cavity or tendon sheath was scored 10, whereas a tumor that was 70% of that volume was scored 7, a tumor that was twice the volume of the maximally distended synovial cavity or tendon sheath was scored 20, etc.

Finally, a passive range of motion (ROM) assessment, standardized according to American Medical Association disability criteria and uses standard goniometers [11], was completed as an objective measure of physical functioning.

#### Assessments

**Fig. 1:**
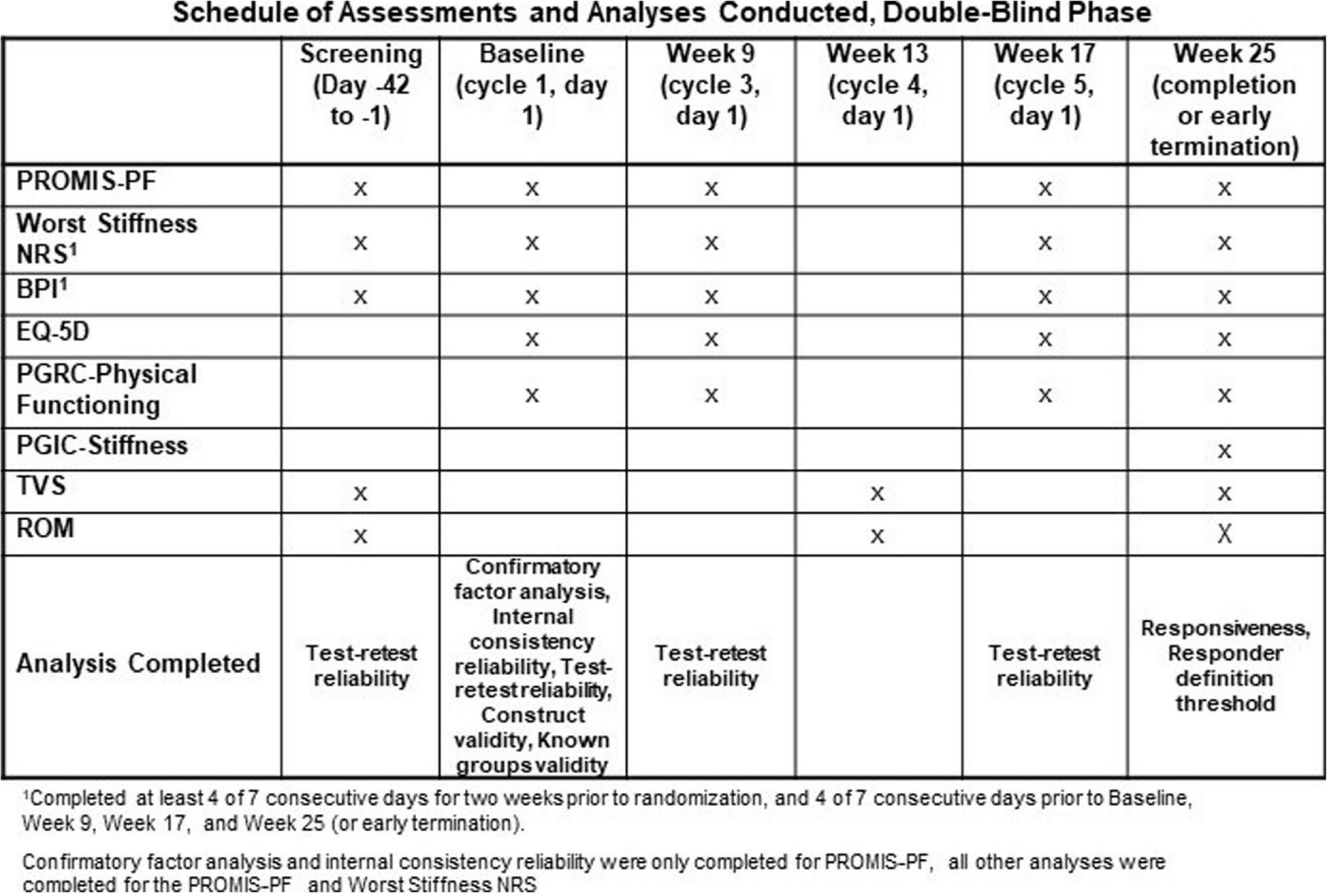
Schedule of Assessments, Double-Blind Phase

All PROs were completed via electronic handheld device in the local language of the study participant. The assessment time points for these analyses focus on the double-blind phase and are shown in Fig. 1.

### Statistical analysis

The analytical methods were undertaken to assess item performance, reliability, validity, ability to detect change, and identification of responder definition thresholds for the PROMIS-PF and Worst Stiffness NRS. The January 31, 2018, data cutoff was used for these analyses. Descriptive statistics were used to characterize the socio-demographic and clinical characteristics of the sample, as well as the Baseline and Week 25 PROMIS-PF and Worst Stiffness NRS scores. Confirmatory factor analysis (CFA) was conducted for the PROMIS-PF LE and UE item sets to confirm that the 15 PF candidate items comprised a single underlying factor in patients with TGCT. Model fit was assessed with comparative fit index (CFI), root mean square error approximation (RMSEA), and average weighted correlation residuals (SRMR). CFI > 0.95 was considered a good fit, as well as RMSEA < 0.05 and SRMR < 0.08.

Internal consistency reliability of the PROMIS-PF LE and UE item sets was assessed at Baseline to determine the extent to which individual items in the instrument were related to one another. Cronbach’s alphas ≥0.70 are considered acceptable [15]. Test-retest reliability of the PROMIS-PF and the Worst Stiffness NRS was evaluated to assess the reproducibility of scores when patients were presumed to be stable. Specifically, the test-retest reliability of the PROMIS-PF was assessed among all subjects between Screening and Baseline, and from Week 9 to 17 among subjects with no change on the PGRC – Physical Functioning. For the Worst Stiffness NRS, data from all subjects between each of 2 consecutive days from Day − 1 to Day-7 (e.g., Day − 2 vs Day − 3, Day − 3 vs. Day − 4) was used. Weekly scores (i.e., 7-day average estimates) for Baseline compared with Screening were also analyzed, as well as from Week 9 to 17 among subjects with no change on the PGIC – Stiffness measure. Intraclass correlation coefficients (ICC) were calculated. The ICC ranges from 0.00–1.00; an ICC ≥0.70 among stable subjects is considered acceptable to demonstrate test-retest reliability [16].

Construct validity of the PROMIS-PF and Worst Stiffness NRS was evaluated at Baseline by examining the relationships with the BPI Worst Pain NRS, EQ-5D, TVS, and ROM. All relationships were assessed via the Spearman’s rank-order correlation coefficient. Cohen’s conventions were used to interpret the absolute value of the correlation results, where a correlation > 0.5 is large, 0.3 to ≤0.5 is moderate, 0.1 to < 0.3 is small, and < 0.1 is insubstantial [3]. It was hypothesized that both measures would have large correlations with BPI Worst Pain NRS, and moderate correlations with each other. It was hypothesized that the correlations with the EQ-5D mobility, self-care, usual activities, and pain/discomfort items would be moderate to large.

To assess known-groups validity, which is the extent to which scores from an instrument are different for groups of participants that differ on a relevant clinical or other indicator, the PROMIS-PF and Worst Stiffness NRS were analyzed by levels of pain (no pain, mild, moderate, and severe categories), TVS (small, medium, and large categories), PF limitation (no limitation, low, medium, and high categories), and stiffness (no stiffness, low, medium, and high categories). Mean scores for the PROMIS-PF and Worst Stiffness NRS were compared for each of the groups using analysis of covariance (ANCOVA) (PROC GLM) at Baseline, controlling for age, gender, race, and body mass index (BMI).

A responsiveness analysis of the PROMIS-PF and Worst Stiffness NRS item was completed to evaluate the instruments’ ability to detect changes in participants who had an established change in clinical status. The association between changes in the scores on the PROMIS-PF and Worst Stiffness NRS from Baseline and Week 25 with change scores on the PGRC – Physical Functioning for PROMIS-PF, and PGIC – Stiffness for the Stiffness NRS, and tumor response status (complete response, partial response, progressive disease, and stable disease) defined by RECIST 1.1 response criteria and TVS for both measures, were examined.

Methods to establish the responder definition threshold included triangulation of anchor- and distribution-based analyses. Anchor-based methods are preferred by the FDA for interpretation of PRO scores [8] and were considered the primary analysis. The anchor for the PROMIS-PF was a change in PGRC-Physical Functioning from Baseline to Week 25. Improvement of “-1” was defined as a change in response in any of the following ways: Extremely to Severely; Severely to Somewhat; Somewhat to A little; or A little to Not at all. The mean change in the PROMIS-PF scale observed in the small improvement group (“-1”) was examined as a key anchor-based indicator of a responder. The anchor for Worst Stiffness NRS was change in PGIC-Stiffness from Baseline to Week 25. The mean change score among patients who reported that they were “a little improved” was examined as a key anchor-based indicator of a responder. Distribution-based analyses included the 0.50 and 0.30 baseline SD, as well as one standard error of measurement (SEM). Empirical cumulative distribution function (eCDF) curves were generated for the PROMIS-PF and Worst Stiffness NRS. The eCDF is a continuous (both positive and negative) presentation of the change scores from Baseline to Week 25 on the X-axis and a cumulative proportion of patients with that level of score change on the Y-axis.

## Results

A summary of patients’ baseline demographic and disease characteristics for ENLIVEN are shown in Table 1. The mean ± SD age was 44.5 years ±13.35 years with a range of 18 years to 79 years. More subjects were female (*n* = 71, 59.2%) with the majority identifying as white (*n* = 106, 88.3%). Most tumors were in the lower extremities (*n* = 110, 91.7%), most commonly the knee (*n* = 73, 60.8%), and ankle (*n* = 21, 17.5%). Based on responses available from 94 subjects, the most disturbing symptom was reported as ‘difficulty with everyday activities’ (*n* = 54, 57.4%), followed by ‘pain’ (*n* = 25, 26.6%) and ‘stiffness’ (*n* = 15, 6.0%).

**Table 1:** Demographic and Baseline Characteristics (ITT Analysis Set)

	Randomized to Placebo(***N*** = 59)	Randomized to Pexidartinib(***N*** = 61)	Total(N = 120)
**Age (yrs)**			
Mean	44.3	44.6	44.5
SD	13.58	13.23	13.35
Median	45.0	44.0	44.5
Minimum	18	22	18
Maximum	79	75	79
**Sex**			
Male	23 (39.0)	26 (42.6)	49 (40.8)
Female	36 (61.0)	35 (57.4)	71 (59.2)
**Race**			
White	54 (91.5)	52 (85.2)	106 (88.3)
Black or African American	1 (1.7)	3 (4.9)	4 (3.3)
Asian	2 (3.4)	1 (1.6)	3 (2.5)
American Indian or Alaskan Native	0	2 (3.3)	2 (1.7)
Native Hawaiian or Other Pacific Islander	2 (3.4)	2 (3.3)	4 (3.3)
Other/Specify	0	1 (1.6)	1 (0.8)
Multi-Racial	0	1 (1.6)	1 (0.8)
**Ethnicity**			
Hispanic/Latino	8 (13.8)	9 (15.5)	17 (14.7)
Not Hispanic or Latino	50 (86.2)	49 (84.5)	99 (85.3)
Missing	1	3	4
**Height (cm)**			
n	56	59	115
Mean	170.64	170.37	170.50
SD	10.501	9.417	9.917
Median	170.00	171.00	170.00
Minimum	152.0	149.0	149.0
Maximum	198.0	195.0	198.0
**Weight (kg)**			
n	59	61	120
Mean	82.11	83.33	82.73
SD	20.122	23.830	22.001
Median	81.00	80.00	80.65
Minimum	48.0	43.0	43.0
Maximum	134.6	151.0	151.0
**Geographic region**			
US Region	22 (37.3)	23 (37.7)	45 (37.5)
Ex-US Region	37 (62.7)	38 (62.3)	75 (62.5)
**Time from Diagnosis to Randomization (days)**			
Mean	1427.8	2449.3	1947.1
SD	1495.24	3098.69	2488.77
Median	926.0	1456.0	1272.0
Minimum	42	15	15
Maximum	8088	14,912	14,912
**PVNS/ GCT-TS**			
PVNS	53 (89.8)	52 (85.2)	105 (87.5)
GCT-TS	6 (10.2)	9 (14.8)	15 (12.5)
Both	0	0	0
**Extremity Involvement**			
Upper	5 (8.5)	5 (8.2)	10 (8.3)
Shoulder	1 (1.7)	1 (1.6)	2 (1.7)
Elbow	0	1 (1.6)	1 (0.8)
Wrist	2 (3.4)	2 (3.3)	4 (3.3)
Hand	0	0	0
Finger	1 (1.7)	0	1 (0.8)
Spine	1 (1.7)	1 (1.6)	2 (1.7)
Lower	54 (91.5)	56 (91.8)	110 (91.7)
Hip	7 (11.9)	6 (9.8)	13 (10.8)
Knee	39 (66.1)	34 (55.7)	73 (60.8)
Ankle	7 (11.9)	14 (23.0)	21 (17.5)
Foot	1 (1.7)	2 (3.3)	3 (2.5)
Toe	0	0	0
**Most Disturbing Symptom**			
Pain	13 (27.7)	12 (25.5)	25 (26.6)
Stiffness	6 (12.8)	9 (19.1)	15 (16.0)
Difficulty with Everyday Activities	28 (59.6)	26 (55.3)	54 (57.4)
Missing	12	14	26
**Global Rating of Concept**			
**How Much has Tumor Limited Physical Functioning**			
Not at All	4 (8.5)	2 (4.3)	6 (6.4)
A Little	10 (21.3)	17 (36.2)	27 (28.7)
Somewhat	23 (48.9)	18 (38.3)	41 (43.6)
Severely	9 (19.1)	9 (19.1)	18 (19.1)
Extremely	1 (2.1)	1 (2.1)	2 (2.1)
Missing	12	14	26

Individual unidimensional CFA models were fit for the nine PROMIS-PF items that overlapped between the UE and LE scales, and the 13 PROMIS-PF lower extremity items. The model could not be estimated for the 11 PROMIS-PF UE items, as there were only 7 participants with UE tumors with PROMIS-PF data available. Data from 104 LE and UE subjects were included for the model that included the 9 items that overlapped across tumor location, the factor loadings ranged from 0.609–0.881, with the exception of item PFA16R1 (“Are you able to dress yourself, including tying shoelaces and buttoning up your clothes?”), which had a factor loading of 0.394. The model showed moderate fit with CFI and RMSEA values (0.874 and 0.155, respectively) and an SRMR of 0.063. Post-hoc analyses revealed that removing PFA16R1 did not substantially improve the fit of the model. Data from 97 LE subjects were included for the model that included the 13 PROMIS-PF LE items, the factor loadings ranged from 0.626–0.840, with the exception of item PFA12 (“Are you able to push open a heavy door?”), which had a factor loading of 0.425. The model showed moderate fit with CFI and RMSEA values (0.804 and 0.159, respectively) and an SRMR of 0.069. Post-hoc analyses revealed that removing PFA12 did not substantially improve the fit of the model.

In analyses that included 93 and 7 subjects, respectively, Cronbach’s alphas were 0.93 and 0.91 for the PROMIS-PF LE and UE items, demonstrating that the items are highly related to each other and show good internal consistency reliability. The test-retest reliability ICC for the PROMIS-PF was 0.80 among all patients (*n* = 101) during the screening period; it was 0.88 among stable patients (*n* = 33) from Weeks 9–17, defined as those that were stable on the PGRC-Physical Functioning item during this period. When the test-retest reliability of the Worst Stiffness NRS was evaluated on a daily basis (*n* = 84 to 108) (e.g., Day − 7 to Day − 6), the ICCs ranged from 0.81–0.90. From Baseline to Week 25 the ICC was 0.76 among stable patients (*n* = 22) (those whose PGIC-Stiffness scores did not change), and when evaluated as a weekly score (*n* = 29) (Day − 14 to Day − 8 compared to Baseline) the ICC was 0.94.

Construct validity of the PROMIS-PF was supported by a moderate correlation with the BPI (− 0.52) and moderate to strong correlations with the pain/discomfort, mobility, and usual activities items of the EQ-5D (− 0.48, − 0.63, and − 0.67, respectively). Construct validity of the Worst Stiffness NRS was supported by a strong correlation with the BPI (0.83) and moderate correlations with the pain/discomfort, mobility, and usual activities items of the EQ-5D (0.47, 0.37, and 0.31, respectively) (Table 2). The correlations with more clinical measures (such as tumor volume score) were weaker, ranging from − 0.06 to 0.35.

**Table 2:** Construct Validity: Spearman Correlations of the PROMIS-PF and Worst Stiffness NRS item with Other Related Measures (Baseline)

Measures	PROMIS-PF			Worst Stiffness		
	Missing N	N	Corr.	Missing N	N	Corr.
PROMIS-PF	–	–	–	6	114	−0.45***
Worse Stiffness NRS	6	114	−0.45***	–	–	–
BPI	6	114	−0.52***	6	114	0.83***
EQ-5D-5L						
Mobility	19	101	−0.63***	20	100	0.37***
Self-care	19	101	−0.46***	20	100	0.30**
Usual activities	19	101	−0.67***	20	100	0.31**
Pain/discomfort	19	101	−0.48***	20	100	0.47***
Anxiety/depression	19	101	−0.23*	20	100	0.28**
Index Score	19	101	0.62***	20	100	−0.54***
VAS	19	101	0.47***	20	100	−0.21*
Tumor Volume Score	5	115	−0.06	18	112	0.08
Range of Motion measurement	4	116	0.35***	17	113	−0.31***

*p < 0.05; **p < 0.01; ***p < 0.001

Known-groups validity of the PROMIS-PF and Worst Stiffness NRS was strongly supported when evaluated by pain level (all *p*-values < 0.05) (Table 3). PROMIS-PF scores differed among subjects categorized by stiffness level, and Worst Stiffness NRS scores differed among subjects categorized by varying degree of physical function limitations. TVS did not provide evidence of known-groups validity, which is consistent with the concurrent validity findings of a lower correlation with TVS and the rationale for including the PROs as an endpoint in clinical trials.

**Table 3:** Known-Groups Validity: PROMIS-PF and Worst Stiffness NRS

		**Pain Level**^**a**^							**Overall** **F-value** **(*P-*value)**		***P-*value**^**b**^
		**Mild 1–4.9**		**Moderate 5–6.9**		**Severe > 7**					
	**Missing N**	**N**	**LS mean (SE)**	**N**	**LS mean (SE)**	**N**	**LS mean (SE)**				
**PROMIS Physical Functioning**											
All Tumors	10	39	39.85 (1.44)	41	36.64 (1.51)	30	33.74 (1.51)			3.87 (0.0002)	1: 0.0218 / 2:< 0.0001 / 3: 0.0750
Knee Tumors	7	26	39.27 (1.69)	22	36.44 (1.86)	18	33.54 (1.67)			2.39 (0.0196)	1: 0.1669 / 2: 0.0025 / 3: 0.2480
Other (Non-Knee) Lower Extremity Tumors	3	8	45.14 (3.59)	16	42.46 (2.99)	10	38.13 (2.92)			2.51 (0.0412)	1: 0.5264 / 2: 0.0713 / 3: 0.2444
**Worst Stiffness NRS**											
All Tumors	8	39	4.09 (0.31)	42	6.21 (0.32)	31	7.79 (0.32)			21.96 (< 0.0001)	1:< 0.0001 / 2:< 0.0001 / 3:< 0.0001
Knee Tumors	5	26	4.13 (0.39)	23	5.89 (0.42)	19	7.72 (0.38)			11.76 (< 0.0001)	1:< 0.0001 / 2:< 0.0001 / 3:< 0.0001
Other (Non-Knee) Lower Extremity Tumors	3	8	3.13 (0.72)	16	5.89 (0.60)	10	7.55 (0.58)			10.67 (< 0.0001)	1:< 0.0001 / 2:< 0.0001 / 3: 0.0106
		**Tumor Volume Score**^**a**^								**Overall** **F-value** **(*P-*value)**	***P-*value**^**b**^
		**Small < 5**		**Medium 5–10**		**Large > 10**					
	**Missing N**	**N**	**LS mean (SE)**	**N**	**LS mean (SE)**	**N**	**LS mean (SE)**				
**PROMIS Physical Functioning**											
All Tumors	9	41	37.11 (1.75)	33	37.28 (1.55)	37	36.64 (1.72)			1.19 (0.3047)	4: 0.9923 / 5: 0.9397 / 6: 0.9031
Knee Tumors	6	33	36.89 (1.97)	23	35.77 (1.75)	11	36.64 (2.43)			0.87 (0.5680)	4: 0.7806 / 5: 0.9939 / 6: 0.9312
Other (Non-Knee) Lower Extremity Tumors	3	6	36.68 (4.07)	7	40.37 (3.82)	21	39.97 (2.96)			1.35 (0.2681)	4: 0.5196 / 5: 0.5144 / 6: 0.9881
**Worst Stiffness NRS**											
All Tumors	10	42	5.63 (0.59)	32	5.99 (0.53)	36	6.00 (0.59)			0.53 (0.8637)	4: 0.7424 / 5: 0.7086 / 6: 0.9995
Knee Tumors	6	34	5.93 (0.69)	23	6.32 (0.62)	10	6.00 (0.90)			0.37 (0.9558)	4: 0.7932 / 5: 0.9967 / 6: 0.9375
Other (Non-Knee) Lower Extremity Tumors	4	6	7.55 (1.37)	6	7.14 (1.32)	21	6.87 (1.00)			0.60 (0.7472)	4: 0.9358 / 5: 0.7776 6: 0.9577
		**Stiffness Level**^**a**^								**Overall F-value** **(*P-*value)**	***P-*value**^**b**^
		**Low 1–4.9**		**Medium 5–6.9**			**High > 7**				
	**Missing N**	**N**	**LS mean (SE)**	**N**	**LS mean (SE)**	**N**	**LS mean (SE)**				
**PROMIS Physical Functioning**											
All Tumors	10	34	39.77 (1.51)	48	36.48 (1.49)		28	34.03 (1.58)		3.21 (0.0013)	7: 0.0212 / 8: 0.0002 / 9: 0.1436
Knee Tumors	7	20	38.21 (1.82)	30	36.42 (1.68)		16	33.14 (1.94)		1.73 (0.0980)	7: 0.4843 / 8: 0.0245 / 9: 0.1528
Other (Non-Knee) Lower Extremity Tumors	3	10	47.41 (2.92)	14	40.53 (2.55)		10	39.21 (2.46)		4.66 (0.0017)	7: 0.0073 / 8: 0.0033 / 9: 0.7984
		**Physical Functioning Limitation Level**^**a**^								**Overall** **F-value** **(*P-*value)**	***P-*value**^**b**^
		**No Limitation > 45**		**Low 35–45**			**Medium 30–35**	**High < 30**			
	**Missing N**	**N**	**LS mean (SE)**	**N**	**LS mean (SE)**	**N**	**LS mean (SE)**	**N**	**LS mean (SE)**		
**Worst Stiffness NRS**											
All Tumors	10	13	4.62 (0.67)	62	5.52 (0.49)	28	6.32 (0.50)	7	7.31 (0.82)	1.90 (0.0481)	10: 0.4194 11: 0.0459 12: 0.0220 13: 0.2639 14: 0.1048 15: 0.6305
Knee Tumors	7	7	5.07 (0.89)	39	5.68 (0.63)	18	6.24 (0.59)	2	7.79 (1.45)	0.90 (0.5469)	10: 0.8758 11: 0.5640 12: 0.3940 13: 0.7772 14: 0.5292 15: 0.7645
Other (Non-Knee) Lower Extremity Tumors	3	4	5.21 (1.13)	18	7.29 (0.84)	9	8.59 (1.10)	3	9.85 (1.50)	2.18 (0.0650)	10: 0.2141 11: 0.0363 12: 0.0369 13: 0.4281 14: 0.2414 15: 0.7354

^a^Categories were determined based on the distribution of scores and clinical relevance

^b^General linear model (PROC GLM) controlling for age, gender, race, and BMI. LS = least squared means; SE = standard error

Pairwise comparisons between LS means were performed using Scheffe’s test adjusting for multiple comparisons

1 = mild vs. moderate; 2 = mild vs. severe; 3 = moderate vs. severe; 4 = small vs. medium; 5 = small vs. large; 6 = medium vs. large; 7 = low vs. medium; 8 = low vs. high; 9 = medium vs. high; 10 = no limitation vs. low; 11 = no limitation vs. medium;12 = no limitation vs. high; 13 = low vs. medium; 14 = low vs. high; 15 = medium vs. high

Responsiveness of the PROMIS-PF and Worst Stiffness NRS item was supported by evaluating change scores between Baseline and Week 25 among different levels of change in PGRC-Physical Functioning and PGIC-Stiffness (overall F values < 0.001) (Table 4). Analyses by tumor responder status (RECIST 1.1 criteria) and tumor volume responder status showed trends in the expected direction to support responsiveness, but were not statistically significant (data not shown).

**Table 4:** Responsiveness of the PROMIS-PF and Worst Stiffness NRS from Baseline to Week 25

		**Change in PGRC-Physical Functioning**						**Overall** **F-value** **(*P-*value)**^**1**^	***P-*value**^**2**^
		**Worsened** **(△ + 1 or greater)**		**No change**		**Improved** **(△ − 1 or lower)**			
	**Missing N**	**N**	**LS mean (SE)**	**N**	**LS mean (SE)**	**N**	**LS mean (SE)**		
PROMIS Physical Function	64	9	−4.21 (2.46)	20	−1.35 (1.91)	27	5.05 (2.12)	3.93 (0.0002)	1: 0.2773 / 2:< 0.0001 / 3: 0.0002
		**Change in PGRC-Physical Functioning**							**Overall F-value (*P-*value)**^**1**^
		**Worsened (△ + 1 or greater), No change**				**Improved (△ − 1 or lower)**			
	**Missing N**	**N**	**LS mean (SE)**			**N**	**LS mean (SE)**		
PROMIS Physical Function	64	29	−1.66 (1.94)			27	5.95 (2.09)		3.86 (0.0003)
		**Change in PGIC-Stiffness**						**Overall F-value** **(*P-*value)**^**1**^	***P-*value**^**2**^
		**Worsened** **(△ − 1 or lower)**		**No change**		**Improved** **(△ + 1 or greater)**			
	**Missing N**	**N**	**LS mean (SE)**	**N**	**LS mean (SE)**	**N**	**LS mean (SE)**		
Worst Stiffness NRS	74	9	−0.15 (1.04)	14	−2.53 (1.13)	23	−4.36 (0.97)	4.75 (0.0001)	1: 0.0367 / 2:< 0.0001 / 3: 0.0434
		**Change in PGIC-Stiffness**							**Overall F-value (*P-*value)**^**1**^
		**Worsened (△ − 1 or lower), No change**				**Improved (△ + 1 or greater)**			
	**Missing N**	**N**	**LS mean (SE)**			**N**	**LS mean (SE)**		
Worst Stiffness NRS	**74**	**23**	−1.06 (1.08)			23	−3.93 (1.04)		3.80 (0.0010)

^1^General linear model (PROC GLM) controlling for baseline score, age, gender, race, and tumor location

^2^Pairwise comparisons between LS means were performed using Scheffe’s test adjusting for multiple comparisons

1 = worsened vs. no change; 2 = worsened vs. improved; 3 = no change vs. improved

**Fig. 2:**
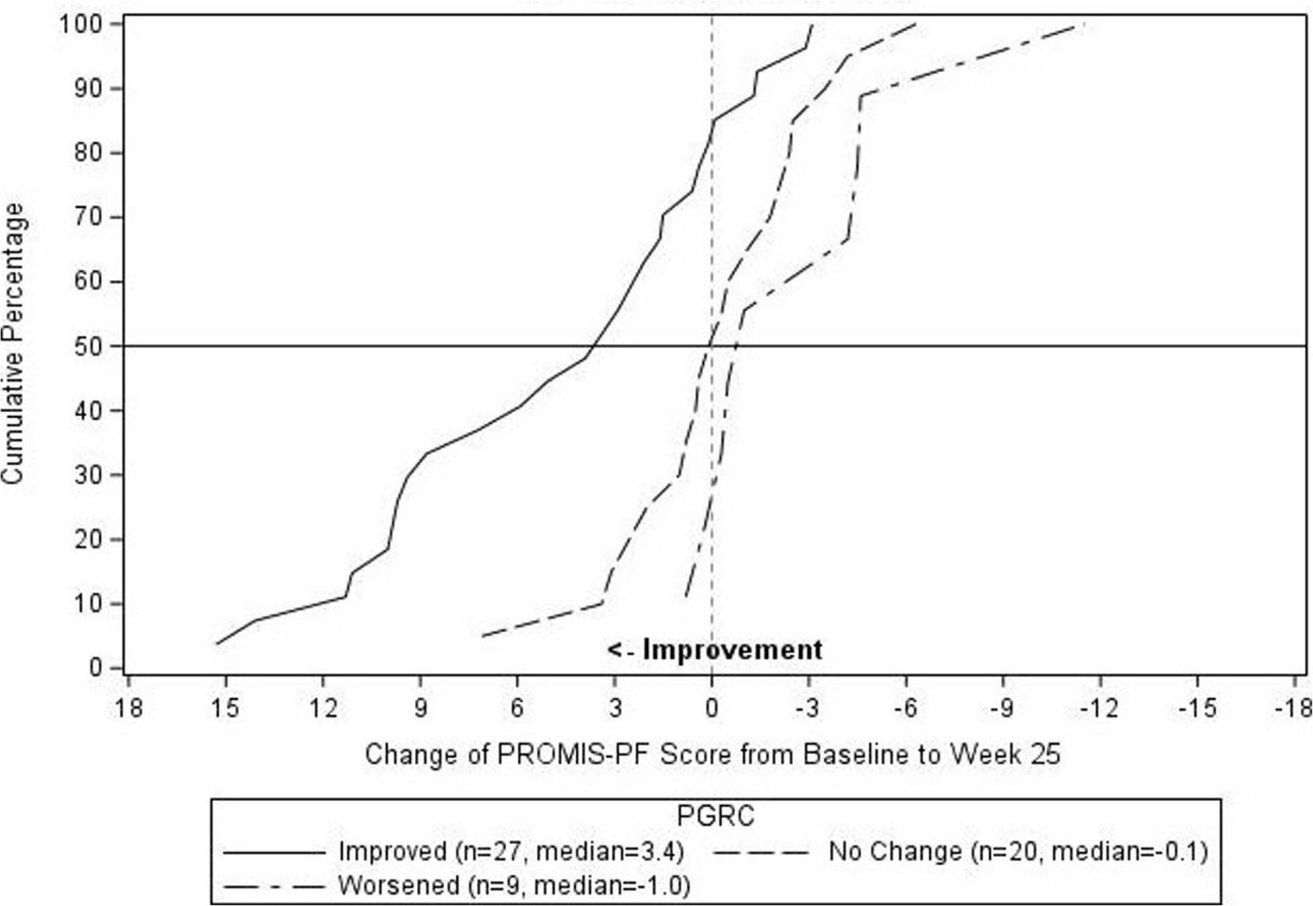
Empirical Cumulative Distribution Function of PROMIS-PF by PGRC-Physical Functioning

**Fig. 3:**
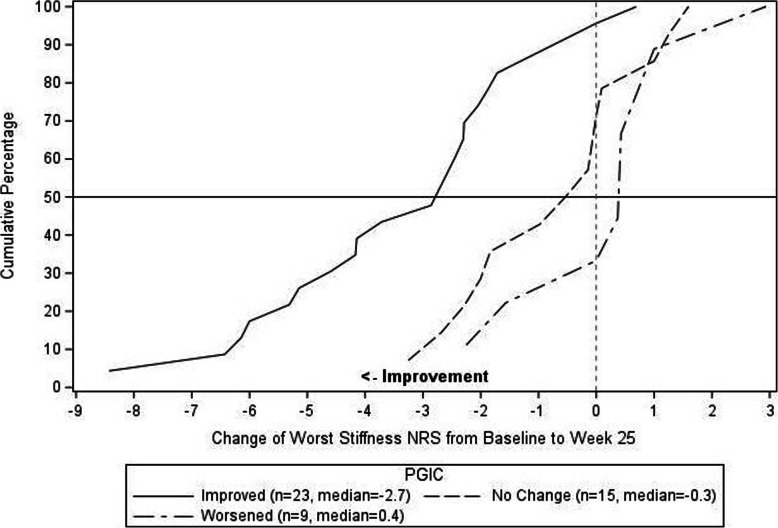
Empirical Cumulative Distribution Function of Worst Stiffness NRS by PGIC-Stiffness

Anchor based analyses demonstrated that improvement of “-1” on the PGRC-Physical Functioning item from Baseline to Week 25, was associated with a least square mean change of 4.04 on the PROMIS-PF. The distribution-based estimates (1/3 SD, 1/2 SD, and 1 SEM) for the PROMIS-PF were 1.85, 2.77, and 2.47, respectively. For the Worst Stiffness NRS, “A little improved” on the perception of stiffness item from Baseline to Week 25, was associated with a least square mean change of − 1.11. The distribution-based estimates (1/3 SD, 1/2 SD, and 1 SEM) for the Worst Stiffness NRS item were 0.61, 0.92, and 0.47, respectively. Triangulation involved examination of the range of estimates and as directed by the FDA PRO guidance, with more consideration allotted to the anchor-based estimates. In selecting a responder definition, the minimum amount of change that is possible on the scale is also considered. This resulted in the responder definition threshold of ≥3 for the PROMIS-PF scale, and ≥ 1 for the Worst Stiffness NRS. The eCDFs are shown in Figs. 2 and 3 for the PROMIS-PF and Worst Stiffness NRS, respectively.

## Discussion

This study provides strong support for the psychometric properties of the PROMIS-PF and Worst Stiffness NRS in the TGCT patient population. Specifically, the internal consistency reliability of the PROMIS-PF was acceptable, the test-retest reliability of both instruments was good, the convergent validity with other PRO measures was adequate, and both instruments were able to differentiate between known groups and detect change over time. In addition, the responder definition thresholds for both instruments was ascertained, which informs the interpretation of meaningful within-person change.

Triangulation of the anchor- and distribution-based methods and the eCDFs resulted in responder definition thresholds of ≥3 for the PROMIS-PF scale, and ≥ 1 for the Worst Stiffness NRS. As seen in the eCDFs there is clear separation between the improved, no change, and worsened groups as the proposed thresholds. For the PROMIS-PF, over 50% of the improved subjects achieved the ≥3 threshold, as compared to roughly 10% of the subjects with no change and none of the subjects that worsened. For the Worst Stiffness NRS, nearly 90% of subjects that improved achieved the ≥1 threshold, as compared to roughly 40% and 30% of the subjects that had no change or worsened, respectively. In the context of clinical practice or research, when a patient is initiating new therapy or undergoing an intervention, these thresholds for change scores can be used to complement the primary clinical outcomes and give clinicians and patients a tangible expectation for measurable benefit.

The responder definition thresholds of ≥3 for the PROMIS-PF scale is consistent with estimates that have been calculated in other patient populations. A minimal important difference range of 4.0–6.0 was estimated by Yost and colleagues [22] using anchor-based methods among a cohort of advanced stage cancer patients. Among patients with rheumatoid arthritis, Hays and colleagues [12] used anchor-based analysis and estimated the minimal important difference to be 2 points (about 0.20 of a standard deviation). Finally, Lee and colleagues [13] used anchor- and distribution-based methods to estimate a range of 1.9–2.2 points as a minimal important difference among patients with knee osteoarthritis.

Although CFA results were not entirely definitive in terms of the item content, it did appear that there was a single common physical functioning latent trait that was defined by each of the respective PROMIS-PF scales. Thus, we proceeded with scoring the PROMIS-PF measures using all available items. This decision was supported by the prior qualitative work in which physicians experienced in the treatment of TGCT indicated that there is a very high degree of heterogeneity in terms of PF impacts in this population [9]. Additionally, patients also exhibited variability in terms of the items that they reported being relevant on an individual basis. Thus, the decision to be more inclusive and retain PROMIS items with lower factor loadings was a conscious one.

As hypothesized and observed in the construct validity analysis, the correlations between the PRO and clinical measures, particularly TVS were weak. The coefficients for PROMIS-PF and Worst Stiffness NRS with TVS were − 0.06 and 0.08, respectively. However, over time, from Baseline to Week 25 the correlations were moderate (− 0.34 and 0.43, respectively). These results support the fact that the PROMIS-PF and Worst Stiffness NRS do measure unique, and patient-relevant outcomes, which are complementary to more morphological tumor response metrics.

A major strength of this study is that it is the first to conduct psychometric validation work on PROs in the TGCT patient population. Generic and orthopedic-related PROs have been used historically among patients with TGCT [19–21], however, none had established content validity or psychometric properties for TGCT. Completion of this current work, and the prior content validity work [9, 10], provides evidence that the PROMIS-PF and Worst Stiffness NRS are fit for purpose [7]. Specifically, this work has demonstrated these measures are appropriate for the patient population and study design, they are valid and reliable concepts that are clinically relevant, and well-defined.

The analytic methods utilized in this study are consistent with the FDA guidance on the use and interpretation of PRO scores in medical product development [8]. However, there is a limitation in the generalizability of the findings to be considered. Only 10 (8.3%) subjects in ENLIVEN had UE tumors. Confidence in the relevance of these results to subjects with UE tumors is limited and replication of these results among a sufficient sample of these subjects would be worthwhile. Further, subgroup analyses such as known-groups validity among LE tumor type were of particular interest given the predominance of knee tumors, however the small sample size (< 10) in many of the criterion groups hindered the interpretability of those results. Another limitation to acknowledge is the considerable amount of post-baseline data that was missing, due mostly to early discontinuations, technical issues with electronic data capture, site and patient compliance, and enrolment being halted just short of the target because of hepatotoxicity. In the context of a psychometric analysis such as this, missing data could impact analyses using post-baseline data, which in this case was the responsiveness analysis and examination of the responder definition thresholds. Despite limited sample size due to missing data, the responsiveness analysis for both the PROMIS-PF and Worst Stiffness NRS were statstitically significant and impressive in the magnitude of difference in score changes between groups. Further, as evidenced by the eCDFs the change groups had clear separation in score changes, giving confidence in use of the data that was available.

## Conclusion

This study is the first to establish the psychometric properties of PRO measures in the TGCT patient population. The evidence provided demonstrates that the PROMIS-PF and Worst Stiffness NRS have good reliability, validity, responsiveness, and provide guidance for their interpretation in this patient population. The PROMIS-PF and Worst Stiffness NRS are well-defined PRO measures that are suitable for use in future trials of therapies for TGCT.

## Data Availability

Not applicable.

## Abbreviations

ANCOVA: Analysis of covariance
BMI: Body mass index
BPI: Brief Pain Inventory
CFA: Confirmatory factor analysis
CFI: Comparative fit index
eCDF: Empirical cumulative distribution function
EQ-5D: EuroQol-5D
ICC: Intraclass correlation coefficients
LE: Lower extremity
NRS: Numeric Rating Scale
PF: PROMIS-Physical Function
PGIC: Patient Global Impression of Change
PGRC: Patient Global Rating of Concept
PRO: Patient-reported outcome
PROMIS: Patient-Reported Outcomes Measurement Information System
QOL: Quality of life
RMSEA: Root mean square error approximation
SD: Standard deviation
SEM: Standard error of measurement
SRMR: Standard root mean squared residual
TGCT: Tenosynovial giant cell tumors
TVS: Tumor volume score
UE: Upper extremity
VAS: Visual analogue scale
